# A Left-Null-Space-Based Massive Access Method for Cell-Free Massive MIMO IoT Systems

**DOI:** 10.3390/s23115285

**Published:** 2023-06-02

**Authors:** Yang Wang, Die Hu

**Affiliations:** Department of Communication Science and Engineering, Fudan University, Shanghai 200082, China; 20210720080@fudan.edu.cn

**Keywords:** cell-free massive multi-input multi-output, MIMO, massive access, interference, pilot contamination, left-null-space, spectral efficiency

## Abstract

Cell-free massive multiple-input multiple-output (MIMO) is a promising technology for the Internet of Things (IoT) because it can increase connectivity and provide considerable energy efficiency (EE) and spectral efficiency (SE). However, pilot contamination caused by pilot reuse severely limits the performance of the system. In this paper, we propose a left-null-space-based massive access method that can significantly reduce interference among users. The proposed method includes three stages: initial orthogonal access, left-null-space-based opportunistic access and data detection of all accessed users. The simulation results show that the proposed method can achieve much higher spectral efficiency than the existing massive access methods.

## 1. Introduction

The Internet of Things (IoT) is envisioned as a promising technology, where highly connected users can share information and coordinate decisions [1]. It has led to revolutionary applications in transportation, smart healthcare, environmental monitoring, smart homes and other scenarios [2]. However, as the number of users connected to the IoT rapidly increases, hyperconnectivity, low latency, high energy efficiency (EE) and spectral efficiency (SE) become new challenges to solve [3]. Cell-free massive multiple-input multiple-output (MIMO) has been proposed in [4] and can meet these requirements. By deploying many access points (APs) in a geographic coverage area and connecting them to a central processing unit (CPU), cell-free massive MIMO can accommodate more users due to coverage probability improvement and interference management [5]. Since the boundaries have disappeared, cell-free massive MIMO can offer much more uniform connectivity, huge energy efficiency and spectral efficiency [6,7,8].

Channel state information (CSI) is important for cell-free massive MIMO systems [9]. However, pilot resources need to be reused among users due to the limitation of coherence duration time, which can lead to the so-called pilot contamination [10]. This phenomenon will significantly reduce channel estimation accuracy and degrade system performance [11]. There are some strategies that have been proposed to limit these negative effects, such as a pilot-precoding-based scheme [12], a channel-estimation-based scheme [13] and a pilot-assignment-based scheme [14]. Although those pilot contamination suppression schemes have achieved certain effects, there is still room for improvement to achieve higher performance of the system, especially in massive access scenarios.

Based on the user density in the network, massive access can be divided into random access and structured access [15]. In [16], random access is classified into two types: contention-based and contention-free. Contention-based random access allows each accessing user to pick a preamble at random from a predefined set and then transmit it to the APs. This method performs well in highly crowded scenarios [17]. In contrast, contention-free random access assigns a dedicated preamble to each user. Therefore, it can eliminate access delays. In [18], the authors improved the random access performance by using the strongest-user collision resolution (SUCR), where only the users with the strongest channel gains are allowed to access the network. In [19], the authors viewed a set of contaminated pilot signals as a graph code on which iterative belief propagation could be performed. This makes it possible to reduce pilot contamination. In [20], the system performance was improved by the averaging of the pilot collision events across the transmission slots for random access. However, if two users in close proximity of each other use the same pilot sequence, the random access usually causes strong pilot contamination and then deteriorates the performance of channel estimation.

A proper pilot assignment can also suppress pilot contamination effects and thus improve the system’s performance in the massive access scenario. Structured access allocates a dedicated pilot resource to every user [21]; thus, contention-free random access can be regarded as a special type of structured access. Structured access is preferable in scenarios where the number of pilots is smaller than the number of users, except in extremely crowded scenarios, similar to the IoT networks [22]. The reason is that the strong pilot contamination might significantly reduce the system’s performance in extremely crowded scenarios. In [15], a structured access framework that focuses on improving pilot assignment schemes was proposed to suppress pilot contamination. A greedy pilot assignment was proposed in [4] that iteratively refines the pilot assignment for the lowest downlink rate user. The authors in [15] proposed a user-group pilot assignment that aims to assign mutually orthogonal pilots to the users served by similar subsets of APs. A K-means-type pilot assignment was proposed in [23] that aimed at maximizing the minimum distance among the pilot sharing users. In [24], a weighted graph was constructed to represent the relationship between the potential strength of pilot contamination between users, which can mitigate pilot contamination by assigning different pilots to connected users with a large weight in a greedy way. The authors of [25] introduced a fractional pilot reuse scheme, where a fraction of users within each cell reuse the same pilot subset across the whole system, while the rest are allocated orthogonal subsets depending on a reuse parameter. However, since all users are allowed to access the network, the pilot assignment method will be less effective due to the limited resources in the crowded scenario.

To reduce inter-user interference, we propose a left-null-space-based massive access method that takes advantage of the resources in the space domain. The idea of reducing interference based on null space has been widely used in the past, especially for the purpose of spatial multiplexing. In [26], a partial nulling scheme was adopted to obtain diversity gains by utilizing degrees of freedom. In addition, Ref. [27] proposed a null-space expansion scheme to mitigate inter-user interference by exploiting the excess degrees of freedom to extend the null-space dimension, which are provided by the massive base station (BS) antenna array. However, most works only focus on the downlink transmission. Specifically, with the given channel matrix, the existing works designed the precoder based on the null space of the channel matrix to mitigate interference. They usually do not consider the channel estimation problem. In this paper, we consider the uplink transmission and focus on the access problem. We need to obtain accurate channel estimation in the case of multi-user interference, which is different from existing null-space-based work. The proposed method includes three stages. In the first initial orthogonal access stage, the users with the strongest channel large-scale fading coefficients are selected for orthogonal access to the network, where the number of these users is the same as that of the pilot sequences. In the second left-null-space-based opportunistic access stage, the users who fall in the left null space of the users in the first stage are selected to access the network. In the last stage, we detect the data of all accessed users. The simulation results demonstrate that, compared with the existing massive access methods, the proposed massive access method can achieve much higher spectral efficiency, especially in crowded scenarios.

The remainder of this paper is organized as follows. Section 2 develops a cell-free massive MIMO IoT system model. Section 3 introduces two traditional massive access methods. Section 4 presents the proposed method, which includes three stages: initial orthogonal access, left-null-space-based opportunistic access and data detection of all accessed users. The performance of three methods is numerically evaluated in Section 5. Finally, the major conclusions and implications are drawn in Section 6.

*Notations:* The superscripts ${}^{T}$, ${}^{H}$ denote transpose and conjugate transpose, respectively; $N\left(H^{T}\right)=\left\{y\in C^{N_{R}\times 1}:y^{T}H=0^{T}\in C^{1\times N_{T}}\right\}$ represents the left null space of a matrix $H\in C^{N_{R}\times N_{T}}$; $I_{n}$ stands for the n×n identity matrix; $N_{C}\left(0,\sigma^{2}I_{n}\right)$ denotes the multivariate circularly symmetric complex Gaussian distribution with zero mean and variance $\sigma^{2}I_{n}$; ${∥x∥}_{2}$ and E{x} denote the Euclidean norm and the expected value of x, respectively.

## 2. System Model

Cell-free massive MIMO was proposed in [4] and can offer a high coverage probability. According to the varying degrees of cooperation between the APs and the CPU, there are four levels of receiver cooperation for cell-free massive MIMO, which can be called Level 4, Level 3, Level 2 and Level 1. Level 4 is a fully centralized network where all APs forward the signals to the CPU without any processing, and all channel estimation and data detection are performed at the CPU. Level 3 relies on the large-scale fading decoding (LSFD) strategy, where every AP locally estimates the channels, obtains the local estimates of the data and then passes them to the CPU for final decoding. Level 2 is a direct simplification of Level 3; the only difference between the two levels is the data detection at the CPU, where Level 2 simply takes the average of the local estimates and Level 3 uses an optimized weighting coefficient vector to maximize the SE. Level 1 is a small-cell network where all the channel estimates and data detection are performed locally at the APs. In this case, there is nothing to exchange between the CPU and the APs. The performances of the aforementioned four levels of receiver cooperation are analyzed and compared in [28], where it is shown that Level 4 can achieve the highest SE. Therefore, in this paper, we focus on Level 4.

**Figure 1:**
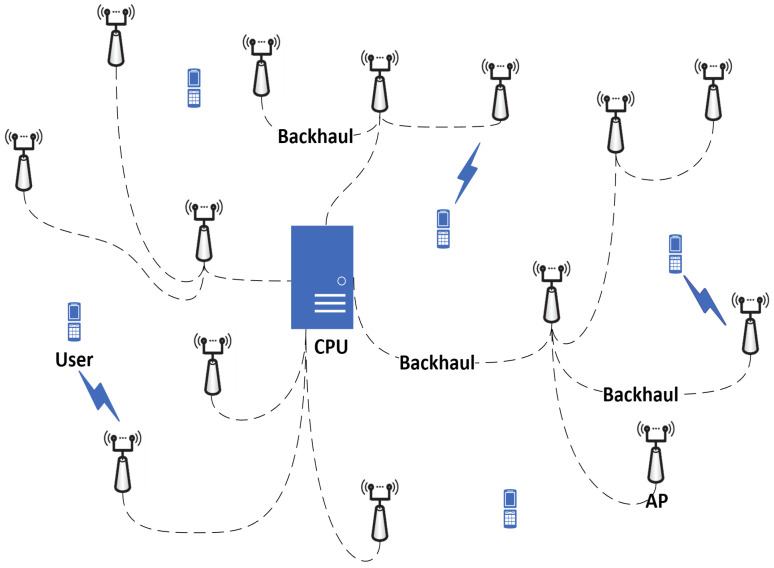
A cell-free massive MIMO IoT system.

Consider a cell-free massive MIMO system consisting of *L* APs equipped with *N* antennas. The APs are connected to a CPU via a backhaul network [4], as illustrated in Figure 1. The users waiting for connection to the system are equipped with a single antenna. All *L* APs directly send the received pilot sequences and data signals to the CPU without processing, and the CPU performs channel estimation and data detection.

The received *n*th vector at the CPU, $y\left(n\right)\in C^{LN\times 1}$, can be expressed as

(1) $$\begin{array}{c} y\left(n\right)=\sum_{k=1}^{K}\sqrt{p_{k}}h_{k}x_{k}\left(n\right)+z\left(n\right) \end{array}$$

where *K* is the number of accessed users, $p_{k}$ denotes the transmit power of the user *k*, $x_{k}\left(n\right)$ denotes the *n*th symbol transmitted by the user *k*, $z\left(n\right)\in C^{LN\times 1}$ is the complex Gaussian noise vector with zero mean and covariance matrix $\sigma^{2}I_{LN}$, $h_{k}={\left[h_{1k}^{T},...,h_{Lk}^{T}\right]}^{T}\in C^{LN\times 1}$ denotes the channel vector between the user *k* and all APs, $h_{lk}\in C^{N\times 1}$ is the channel vector between the user *k* and the AP *l*, which is given by

(2) $$\begin{array}{c} h_{lk}=\beta_{lk}^{1/2}{\tilde{h}}_{lk} \end{array}$$

$\beta_{lk}$ denotes the large-scale fading coefficient that depends on the location and the propagation environment, and ${\tilde{h}}_{lk}∼N_{C}\left(0,I_{N}\right)$ represents the small-scale fading coefficient vector. We assume that $h_{k}$ is constant in a time block of $\tau_{c}$ channel uses [11], where $\tau_{p}$ channel uses are specifically for pilots and the remaining $\tau_{c}-\tau_{p}$ channel uses are for payload data.

After collecting *J* consecutive received vectors, we can obtain

(3) $$\begin{array}{c} Y=\sum_{k=1}^{K}\sqrt{p_{k}}h_{k}x_{k}+Z \end{array}$$

where $Y=\left[y\left(1\right),...,y\left(J\right)\right]\in C^{LN\times J}$, $x_{k}=\left[x_{k}\left(1\right),...,x_{k}\left(J\right)\right]\in C^{1\times J}$ and $Z=\left[z\left(1\right),...,z\left(J\right)\right]\in C^{LN\times J}$.

## 3. Traditional Massive Access Methods

Assume that there are $\tau_{p}$ mutually orthogonal $\tau_{p}$-length pilot sequences $\phi_{1},...,\phi_{\tau_{p}}\in C^{1\times \tau_{p}}$ with $∥\phi_{m}{∥}_{2}^{2}=\tau_{p}$, where $m=1,...,\tau_{p}$. Consider a highly crowded scenario where the total number of the users *U* is much larger than the number of available pilot sequences $\tau_{p}$. Since different users have to reuse the same pilot sequence, how to reduce interference and improve the system’s performance becomes very important. Ref. [23] proposed a K-means-clustering-based pilot assignment method for cell-free massive MIMO systems, which can reduce the effect of the pilot contamination. In this method, the CPU first generates $U/\tau_{p}$ centroids through the K-means clustering algorithm, which can be performed offline. Specifically, $J_{p}$ points and $U/\tau_{p}$ centroids are first arbitrarily generated in the coverage area, where $J_{p}\gg U$. Then, each point is assigned to its nearest centroid. After that, the centroid positions are updated by averaging the points belonging to the respective clusters. Next, all $J_{p}$ points are reassigned to the new centroids. The above assignment is repeated, and the steps are updated until $U/\tau_{p}$ stable clusters are formed. Each cluster has a centroid and comprises at most $\tau_{p}$ users. After the centroids are determined, all *U* users access the network in sequence, where each user finds its closest centroid and joins the corresponding cluster. For any user, if the closest centroid has been selected by other $\tau_{p}$ users, then it tries to join the next closest centroid, and repeats this procedure until it succeeds in joining a cluster. After all users find their clusters, $\tau_{p}$ orthogonal pilot sequences are arbitrarily assigned to the users in the same cluster.

Different from the method where all users are allowed to access the system in [23], another massive access method is based on user selection. That is, according to the users’ channel conditions, such as large-scale fading coefficients, the CPU selects *K* users from *U* users to access the system [18]. More specifically, let ${\bar{\beta }}_{k}$ denote the average large-scale fading coefficient from the user *k* to all APs, where ${\bar{\beta }}_{k}≜\frac{1}{L}\sum_{l=1}^{L}\beta_{lk}$. *K* users with the largest ${\bar{\beta }}_{k}$ are allowed to access the network. In order to avoid strong interference among the users with good channel conditions, one can assign $\tau_{p}$ pilot sequences to the $\tau_{p}$ users with the best channel conditions and arbitrarily assign the pilot sequences to the remaining $K-\tau_{p}$ users.

After pilot assignment for the accessed users, the CPU performs channel estimation for each user based on the received pilot sequences. Since the number of accessed users *K* is more than $\tau_{p}$, the same pilot sequence may be assigned to several users. Let $S_{k}$ represent the set of users sharing the same pilot sequence with user *k*, including user *k* itself. According to (3), during the training phase, the received pilot matrix at the CPU can be obtained as

(4) $$\begin{array}{c} Y^{p}=\sum_{k=1}^{K}\sqrt{p_{k}}h_{k}\phi_{m_{k}}+Z^{p} \end{array}$$

where $m_{k}\in \left\{1,...,\tau_{p}\right\}$ is the index of the pilot sequence assigned to the user *k*.

Given (4), the least square (LS) estimate of $h_{k}$ can be obtained as

(5) $$\begin{array}{cc} {\hat{h}}_{k}= & \frac{1}{\tau_{p}\sqrt{p_{k}}}Y^{p}\phi_{m_{k}}^{H}\\ = & \frac{1}{\tau_{p}\sqrt{p_{k}}}\left(\sqrt{p_{k}}h_{k}\phi_{m_{k}}+\sum_{i\in S_{k},i\neq k}\sqrt{p_{i}}h_{i}\phi_{m_{k}}+\sum_{j\notin S_{k},j=1}^{K}\sqrt{p_{j}}h_{j}\phi_{m_{j}}+Z^{p}\right)\phi_{m_{k}}^{H}\\ = & h_{k}+\sum_{i\in S_{k},i\neq k}h_{i}+{\bar{z}}_{k} \end{array}$$

where ${\bar{z}}_{k}=\frac{1}{\tau_{p}\sqrt{p_{k}}}Z^{p}\phi_{m_{k}}^{H}$. The third equation follows from the facts that $\phi_{m_{k}}\phi_{m_{k}}^{H}=\tau_{p}$ and $\phi_{m_{j}}\phi_{m_{k}}^{H}=0$ for $m_{j}\neq m_{k}$. Note that the second term $\sum_{i\in S_{k},i\neq k}h_{i}$ in the third equation may cause so-called pilot contamination and degrade the system performance. Here we only consider LS channel estimator because it has low complexity and requires no prior statistical information [29].

Given ${\hat{h}}_{k}$, the achievable spectral efficiency of the user *k* can be calculated by [28]

(6) $$\begin{array}{c} {\mathrm{SE}}_{k}=\left(1-\frac{\tau_{p}}{\tau_{c}}\right)E\left\{{\log_{2}(1+\mathrm{SINR}}_{k})\right\} \end{array}$$

where the signal-to-interference-and-noise ratio (SINR) is given by

(7) $$\begin{array}{c} {\mathrm{SINR}}_{k}=\frac{p_{k}{\left|v_{k}^{H}h_{k}\right|}^{2}}{\sum_{i=1,i\neq k}^{K}p_{i}{\left|v_{k}^{H}h_{i}\right|}^{2}+\sigma^{2}v_{k}^{H}v_{k}} \end{array}$$

and $v_{k}\in C^{LN\times 1}$ is the combining vector for the user *k*. Any received combining vector $v_{k}$ can be adopted in (7). For example, one can let $v_{k}={\hat{h}}_{k}$, i.e., use the simplest maximum ratio (MR) combination.

## 4. The Proposed Massive Access Method

In this section, we propose a null-space-based massive access method for cell-free massive MIMO systems that can significantly reduce interference between users. Similar to [18], we consider a crowded system with *U* users and finally select *K* users to access the system. The proposed massive access method consists of three stages, which are summarized as follows.

1. *Stage 1: Initial orthogonal access*
  (1) Step 1: Select $\tau_{p}$ users from all users and allow them to access the system.
  (2) Step 2: Allocate $\tau_{p}$ orthogonal pilot sequences to the accessed users and estimate their channel vectors.
2. *Stage 2: Left-null-space-based opportunistic access*
  (1) Step 1: Compute the accessed users’ left null space according to the results of the channel estimation in Stage 1 and obtain the bases of the left null space.
  (2) Step 2: Based on the left null space, select $K-\tau_{p}$ users from the remaining $U-\tau_{p}$ users to access the network.
  (3) Step 3: Estimate the channel of the new selected $K-\tau_{p}$ users.
3. *Stage 3: Detect the data of all K-accessed users.*

**Figure 2:**
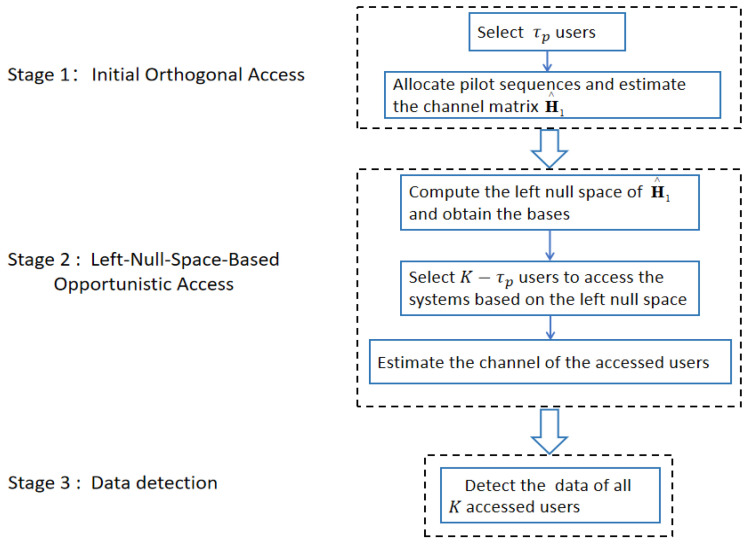
The proposed massive access method.

Figure 2 is a flow diagram of the proposed method. In the proposed method, $\tau_{p}$ users in the first stage can access the system without interference, and $K-\tau_{p}$ users in the second stage can access the system with less interference. The data of all *K* users are detected in the last stage. More details about the three stages are given in Section 4.1, Section 4.2 and Section 4.3, respectively.

### 4.1. Stage 1: Initial Orthogonal Access

In this stage, the CPU allows $\tau_{p}$ users to access the system according to the number of pilot sequences $\tau_{p}$. Different schemes can be adopted to select these users. For example, the simplest way is to randomly select $\tau_{p}$ users from all users. However, such a random selection scheme cannot guarantee the performance of the channel estimation because it may select some users with poor channel conditions. In order to ensure the accuracy of the channel estimation, we adopt the similar user selection method described in Section 3. That is, we select $\tau_{p}$ users based on the strongest channel large-scale fading coefficients and then allocate $\tau_{p}$ orthogonal pilot sequences to them.

Due to the orthogonality of the pilot sequences, according to (5), the channel estimation of the user *k* can be given by

(8) $$\begin{array}{c} {\hat{h}}_{k}=h_{k}+{\bar{z}}_{k} \end{array}$$

where $k=1,...,\tau_{p}$. It can be seen that there is no interference between the users. Furthermore, since all the selected users have good channel conditions, the accuracy of the channel estimation can be guaranteed.

### 4.2. Stage 2: Left-Null-Space-Based Opportunistic Access

To exploit the resources in the spatial domain, we propose an algorithm based on the orthogonal property of the left null space. By taking advantage of the orthogonality between different bases of the left null space, the CPU can distinguish different users in the spatial domain even though they share the same pilot sequence. This is equivalent to adding an additional spatial domain dimension to the original time and frequency domain dimensions, which can significantly reduce the pilot contamination effect.

In this stage, all the remaining $U-\tau_{p}$ users first randomly choose their pilot sequence from the predefined set and then transmit them to the APs. After all of the APs forward the received signals to the CPU, the received pilot matrix at the CPU, $Y_{2}^{p}\in C^{LN\times \tau_{p}}$, can be obtained as

(9) $$\begin{array}{cc} Y_{2}^{p} & =\sum_{i=1}^{U-\tau_{p}}\sqrt{p_{i}}h_{i}\phi_{m_{i}}+Z_{2}^{p}\\ & =\sum_{m=1}^{\tau_{p}}\sum_{i\in P_{m}}\sqrt{p_{i}}h_{i}\phi_{m}+Z_{2}^{p} \end{array}$$

where $P_{m}$ is the set of users sharing the same pilot sequence *m* and $Z_{2}^{p}\in C^{LN\times \tau_{p}}$.

To select $K-\tau_{p}$ users with the least interference from the whole $U-\tau_{p}$ users, the CPU then correlates the received signal with the normalized pilot sequence $\phi_{m}^{H}/\sqrt{\tau_{p}}$ to obtain ${\bar{h}}_{m}\in C^{LN\times 1}$, which is given by

(10) $$\begin{array}{cc} {\bar{h}}_{m} & ≜\frac{1}{\sqrt{\tau_{p}}}Y_{2}^{p}\phi_{m}^{H}\\ & =\sum_{i\in P_{m}}\sqrt{p_{i}\tau_{p}}h_{i}+{\bar{z}}_{m} \end{array}$$

where ${\bar{z}}_{m}=\frac{1}{\sqrt{\tau_{p}}}Z_{2}^{p}\phi_{m}^{H}$. By doing so, users are divided into $\tau_{p}$ groups, where each group of users shares the same pilot sequence.

After that, the CPU computes the left null space $N({\hat{H}}_{1}^{T})$ according to the channel estimations obtained in Stage 1, where ${\hat{H}}_{1}=\left[{\hat{h}}_{1},...,{\hat{h}}_{\tau_{p}}\right]\in C^{LN\times \tau_{p}}$. QR factorization can be used to obtain $N({\hat{H}}_{1}^{T})$, i.e., the right $LN-\tau_{p}$ column of the Q matrix after QR factorization is $N({\hat{H}}_{1}^{T})$. Let $e_{n}$ denote the *n*th column of the $N({\hat{H}}_{1}^{T})$ and define $W_{n}=e_{n}e_{n}^{H}$, where $n=1,...,LN-\tau_{p}$. $e_{n}$ represents the base of the left null space. Different bases are orthogonal to they are all orthogonal to ${\hat{H}}_{1}$. each other and Given $N({\hat{H}}_{1}^{T})$, the CPU next searches for the users who fall in the left null space. Specifically, the CPU computes the following projection as

(11) $$\begin{array}{cc} {\bar{g}}_{n,m}= & W_{n}{\bar{h}}_{m}\\ = & \sum_{i\in P_{m}}\sqrt{p_{i}\tau_{p}}W_{n}h_{i}+W_{n}{\bar{z}}_{m}\\ = & \sqrt{p_{j}\tau_{p}}W_{n}h_{j}+\sum_{i\in P_{m},i\neq j}\sqrt{p_{i}\tau_{p}}W_{n}h_{i}+W_{n}{\bar{z}}_{m} \end{array}$$

If the energy of ${\bar{g}}_{n,m}$ is greater than a certain threshold ε, i.e.,

(12) $$\begin{array}{c} E_{n,m}>\varepsilon \end{array}$$

where $E_{n,m}={∥{\bar{g}}_{n,m}∥}_{2}^{2}$, we consider that there is a user in the left null space. Here we assume that there is at most one user whose channel vector matches the base $e_{n}$. Once (12) happens, the CPU puts this user, whose index is denoted by $j_{\left(n,m\right)}$, into the list $T_{s}$, where $T_{s}$ denotes the set of index values of the users that are allowed to access the system. Note that by adjusting the threshold ε, the CPU can control the number of accessed users in Stage 2.

When (12) occurs, (11) can be further written as

(13) $$\begin{array}{c} {\bar{g}}_{n,j_{\left(n,m\right)}}=\sqrt{p_{j_{\left(n,m\right)}}\tau_{p}}W_{n}h_{j_{\left(n,m\right)}}+W_{n}{\bar{z}}_{m} \end{array}$$

because $W_{n}h_{i}=0$ when users are in different subspaces. Let $g_{n,j_{\left(n,m\right)}}$ be the projection of the true channel of the user $j_{\left(n,m\right)}$, i.e.,

(14) $$\begin{array}{c} g_{n,j_{\left(n,m\right)}}≜W_{n}h_{j_{\left(n,m\right)}} \end{array}$$

Then, the LS estimate of $g_{n,j_{\left(n,m\right)}}$ can be obtained as

(15) $$\begin{array}{cc} {\hat{g}}_{n,j_{\left(n,m\right)}}= & \frac{1}{\sqrt{p_{j_{\left(n,m\right)}}\tau_{p}}}{\bar{g}}_{n,j_{\left(n,m\right)}}\\ = & g_{n,j_{\left(n,m\right)}}+\frac{1}{\sqrt{p_{j_{\left(n,m\right)}}\tau_{p}}}W_{n}{\bar{z}}_{m} \end{array}$$

Based on the above algorithm, the CPU can find the users who are in the left null space of the accessed users in Stage 1. Allowing these users access into the system can greatly reduce inter-user interference. Here, we assume $K-\tau_{p}$ users successfully accessed the system in the end. The proposed left-null-space-based access algorithm is summarized in Algorithm 1.
**Algorithm 1:** Left-Null-Space-Based Access 1The remaining $U-\tau_{p}$ users randomly transmit the pilot sequence to the APs. 2Given the received pilot matrix $Y_{2}^{p}$, the CPU obtains ${\bar{h}}_{m}$ by using (10), where $m=1,...,\tau_{p}$. 3Given ${\hat{h}}_{k}$, compute $N({\hat{H}}_{1}^{T})$ by, e.g., QR factorization. Obtain $e_{n}$ and then $W_{n}=e_{n}e_{n}^{H}$, where $n=1,...,LN-\tau_{p}$. Set $T_{s}=\emptyset$. 4**for** 1 $\leq m\leq \tau_{p}$ **do**( 5  **for** 1 $\leq n\leq LN-\tau_{p}$ **do** 6    Obtain ${\bar{g}}_{n,m}$ according to (11). 7    Calculate the energy of ${\bar{g}}_{n,m}$, i.e., $E_{n,m}={∥{\bar{g}}_{n,m}∥}_{2}^{2}$. 8    **if** $E_{n,m}>\varepsilon$ **then** 9      Put user $j_{\left(n,m\right)}$ into the list $T_{s}$.10    **end**11  **end**12**end**13final

In summary, the proposed massive access method has the following advantages. Firstly, the users accessed in Stage 1 use the orthogonal pilot sequences to access the system; hence, they have no interference and accurate channel estimation. Secondly, due to the orthogonality between $N({\hat{H}}_{1}^{T})$ and ${\hat{H}}_{1}$, users in Stage 2 will cause less interference to users in Stage 1. Finally, since the users accessed in Stage 2 are selected according to the bases and different bases are orthogonal to each other, the pilot contamination effect caused by pilot-sharing users will be significantly reduced in Stage 2. A similar method is to add a space-domain dimension, where users are able to share the same pilot with no interference. By doing so, the number of accessed users can be greatly increased with less interference.

### 4.3. Stage 3: Detect the Data of All *K*-Accessed Users

According to (8) and (15), the estimation of the channel for all accessed users can be obtained. Finally, the CPU can detect the data of all users based on the estimated channel.

For users accessed in Stage 1, we use the SINR expression in (7) to calculate the achievable SE, where the MR combining with $v_{k}={\hat{h}}_{k}$ is used for data detection and ${\hat{h}}_{k}$ is given by (8).

To detect the data of the users accessed in Stage 2, the CPU needs to project the received signal y into the subspace first. The received signal of the user $j_{\left(n,m\right)}$ after projection can be obtained as

(16) $$\begin{array}{cc} y_{j_{\left(n,m\right)}} & =W_{n}\sum_{i=1}^{K-\tau_{p}}h_{i}s_{i}+W_{n}z\\ & =g_{n,j_{\left(n,m\right)}}s_{j_{\left(n,m\right)}}+\sum_{i\in C_{n},i\neq j_{\left(n,m\right)}}g_{n,i}s_{i}+{\tilde{z}}_{j_{\left(n,m\right)}} \end{array}$$

where $C_{n}$ is the set of the users whose channels match the same base $e_{n}$ and ${\tilde{z}}_{j_{\left(n,m\right)}}=W_{n}z$. The second equation follows the fact that $W_{n}h_{i}=0$ when $i\notin C_{n}$. This implies that users in the same group will not cause any interference, that interference only happens between inter-group users and that there are at most $\tau_{p}$ elements in $C_{n}$.

Since $g_{n,j_{\left(n,m\right)}}$ can be viewed as the equivalent channel of user $j_{\left(n,m\right)}$, we replace $h_{j_{\left(n,m\right)}}$ with $g_{n,j_{\left(n,m\right)}}$ and use MR combination i.e., $v_{j_{\left(n,m\right)}}={\hat{g}}_{n,j_{\left(n,m\right)}}$ in (7) to obtain the effective SINR.

## 5. Numerical Results

In this section, we evaluate the uplink performance of our proposed massive access method. We consider a setup with *U* users who are independently and uniformly distributed at random within a square size of 1 km × 1 km and finally select *K* users from them to access the system. *L* = 100 APs are deployed randomly within the square coverage area, and all APs are equipped with half-wavelength-spaced uniform linear arrays with *N* = 4 antennas. We assume that all APs are deployed in urban environments with high user loads, roughly 10 m above the ground. The 3GPP Urban Microcell model in Tab. B.1.2.1-1 [30] with a 2 GHz carrier frequency is used to generate the large-scale propagation conditions, such as pathloss and shadow fading.

(17) $$\begin{array}{c} \beta_{kl}\left[\mathrm{dB}\right]=-30.5-36.7\log_{10}\left(\frac{d_{kl}}{1m}\right)+F_{kl} \end{array}$$

where $d_{kl}$ is the distance between the user *k* and AP *l* and $F_{kl}∼N(0,4^{2})$ is the shadow fading. We present the key simulation parameters in Table 1.

In the simulations, we use “Strongest Users method” [18] and “K-Means method” [23] to denote the methods described in Section 3 and compare them with the proposed method.

**Figure 3:**
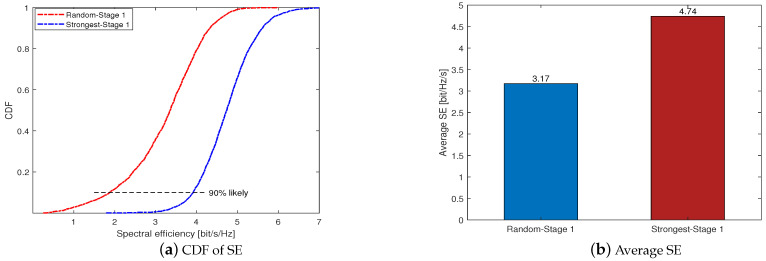
Comparison of different user selection schemes in Stage 1 under *K* = 40 and *U* = 100.

Figure 3 compares the two user selection schemes we proposed in Stage 1 under *K* = 40 and *U* = 100. For the sake of description, we use “Random-Stage 1” and “Strongest-Stage 1” to denote the random selection scheme and the strongest channel large-scale fading coefficients user selection scheme, respectively. Figure 3a shows the cumulative distribution function (CDF) of the SE per user when using an LS estimator and MR combining. As expected, the Strongest-Stage 1 outperforms the Random-Stage 1, where the Strongest-Stage 1 curve is totally on the right of the Random-Stage 1 curve. At the 90% likely SE points, the SE of the the Strongest-Stage 1 and the Random-Stage 1 are 3.91 and 1.87, respectively. Figure 3b shows that the average SE of the Strongest-Stage 1 and the Random-Stage 1 are 4.74 and 3.17, which is consistent with Figure 3a. Since the first scheme ensures the quality of the channel estimation, it can guarantee the accuracy of the obtained left null space. Hence, the strongest channel large-scale fading coefficients user selection scheme performs much better than the random selection scheme, and we use it as the default initial user access scheme in Stage 1 for the following simulation.

**Figure 4:**
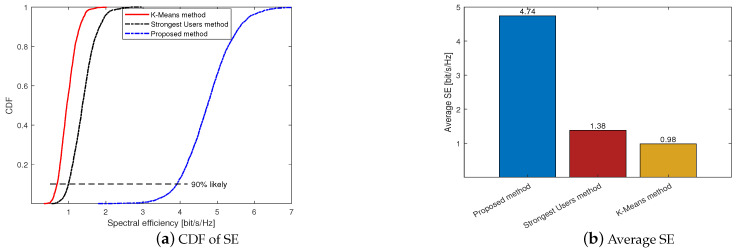
Comparison of different massive access methods under *K* = 40 and *U* = 100.

Figure 4 shows the comparison of the three massive access methods, where we let K=40, U=100 and use an LS channel estimator and MR combining. It can be seen from Figure 4a that the proposed method outperforms the other two. For example, at the 90% likely SE points, the SEs of the proposed method, the Strongest Users method and the K-Means method are 3.91, 0.99 and 0.69, respectively. This means that the proposed method can achieve a higher SE than the other two methods for most users. Figure 4b shows the average SE of the proposed method, Strongest Users method and K-Means method, they are 4.74, 1.38 and 0.98, respectively.

**Figure 5:**
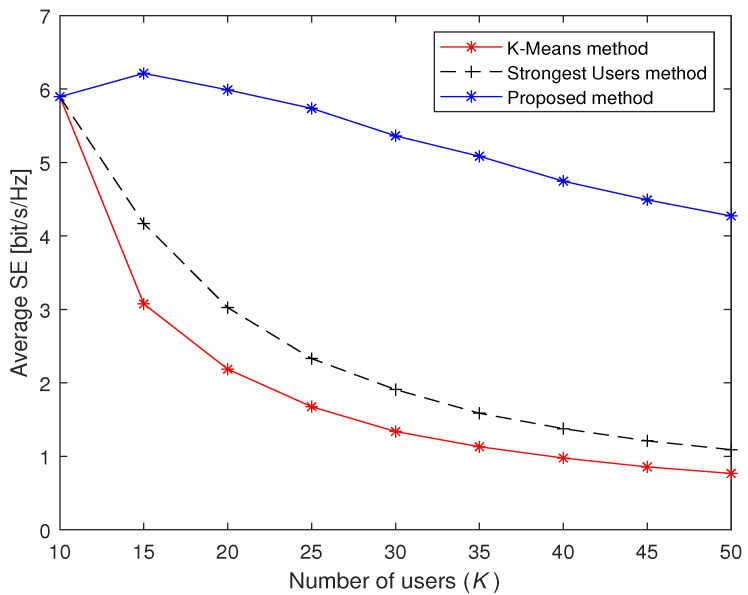
Average SE of three massive access methods under different the number of users *K*.

Figure 5 shows the average SE performance of the three methods under different *K*. We let the number of accessed users in Stage 2 be about one-third of the total users, i.e., $(U-\tau_{p})/3$. It can be seen from Figure 5 that the proposed method has a gentle descent and is always above the other two methods, which means that the performance of the proposed method outperforms the other two methods. When K=50, the average SEs of the proposed method, the Strongest Users method and the K-Means method are 4.27, 1.08 and 0.77, respectively. Note that the average SE is a decreasing function regardless of any massive access methods in the vast majority of intervals. The reason is that the larger number of users can cause stronger inter-user interference, which leads to a rapid reduction in the average SE.

**Figure 6:**
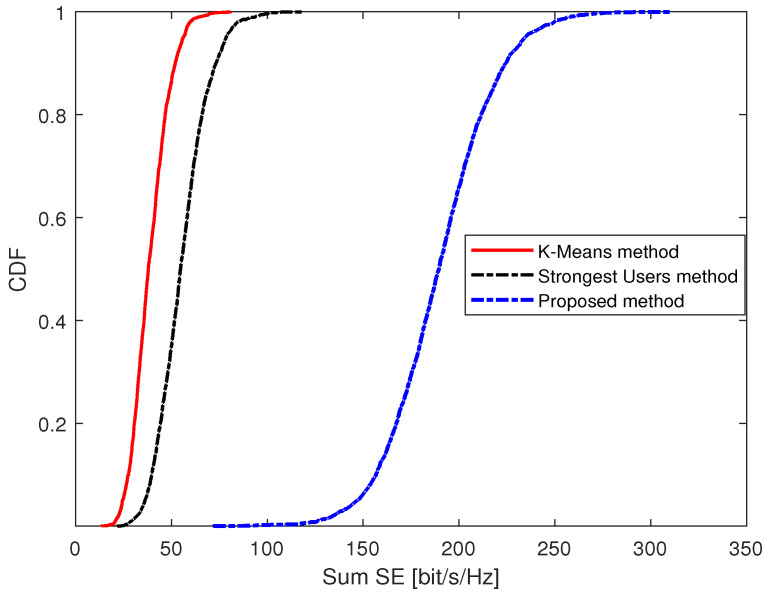
The CDF of the sum SE under different massive access methods under *K* = 40 and *U* = 100.

Figure 6 depicts the CDF of the sum SE over different massive access methods under K=40 and U=100. The result shows that the proposed massive access method outperforms the other two methods, which is echoed by the results in Figure 4. The reason is that the users in our proposed massive access method are less influenced by interference, so it can improve the sum SE compared with the other two methods.

**Figure 7:**
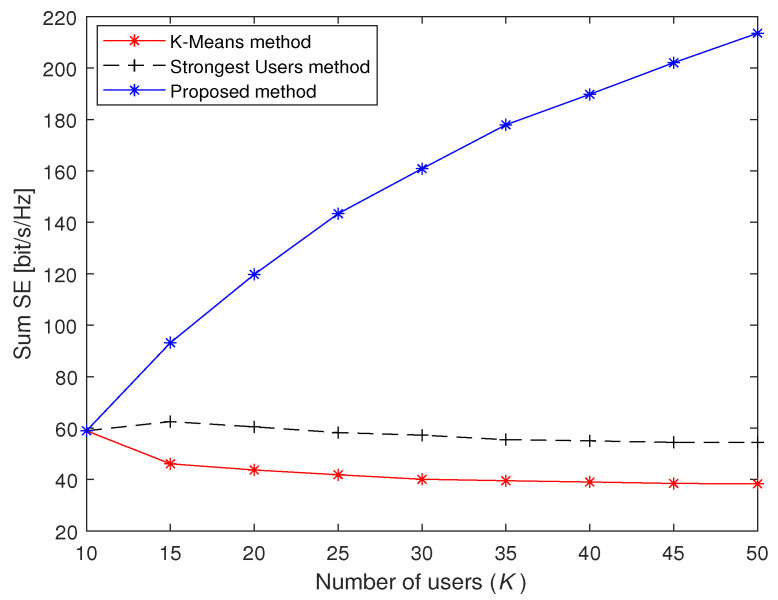
Sum SE of three massive access methods under different the number of users *K*.

Figure 7 shows the sum SE comparison of the three methods under different *K*. The setting of *U* is the same as that in the simulations in Figure 5. When K=40, the sum SE of the proposed method, the Strongest Users method and the K-Means method are 189.77, 55.03 and 39.02, respectively. For the K-Means method, it is clear that the sum SE slowly decreases when the number of access users *K* increases. The Strongest Users method has the same distribution as the K-Means method, but it can achieve a higher sum SE. Compared with the other two methods, the proposed method has much better performance since the curve of the proposed method is an increasing function of *K*. The reason is that the traditional massive access methods are greatly affected by interference; the more users connected to the system, the more obvious this phenomenon is. Since the proposed massive access method can alleviate the impact of multi-user interference through the left-null-space-based access algorithm, the sum SE can be increased in crowded scenarios.

## 6. Conclusions

In this paper, we have proposed a left-null-space-based massive access method for cell-free massive MIMO systems. The key idea of the proposed method is to exploit the space-domain orthogonality. The proposed method first lets some of the users orthogonally access the system, and then the remaining users are given access to the system according to the left null space of the accessed users. The simulation results show that our proposed method performs much better than the existing methods, which can achieve higher SE by suppressing inter-user interference.

Under the current framework, all signal processing is performed on the CPU, which poses some challenges to the CPU’s processing capacity. In addition, the massive data exchange between the AP and the CPU also places a great load on the backhaul network. Therefore, in future research, we will apply this method to a completely distributed small cell network, where all the channel estimations and data detection are performed locally at the APs. Since there are no experimental testbed cases used yet, we are looking forward to verifying these results in future studies. 

## Figures and Tables

**Table 1:** Key simulation parameters.

Parameters	Value
Transmit powers	$p_{k}$ = 100 mW
System bandwidth	*B* = 20 MHz
Noise power	$\sigma^{2}$ = −96 dBm
Coherent bandwidth	100 KHz
Coherent time	20 ms
Total channel uses	$\tau_{c}$ = 200
Pilot sequences	$\tau_{p}$ = 10

## Data Availability

The data that support the findings of this study are available on request from the corresponding author.
